# Epigenetic remodelling of Fxyd1 promoters in developing heart and brain tissues

**DOI:** 10.1038/s41598-022-10365-y

**Published:** 2022-04-19

**Authors:** Mariella Cuomo, Ermanno Florio, Rosa Della Monica, Davide Costabile, Michela Buonaiuto, Teodolinda Di Risi, Giulia De Riso, Antonella Sarnataro, Sergio Cocozza, Roberta Visconti, Lorenzo Chiariotti

**Affiliations:** 1 Department of Molecular Medicine and Medical Biotechnology, University of Naples “Federico II”, 80131 Naples, Italy; 2 Department of Medicine, University of California, San Diego UCSD, Gilman Dr, La Jolla, CA 95000 USA; 3 CEINGE-Biotecnologie Avanzate, Via Gaetano Salvatore, 486, 80145 Naples, Italy; 4 SEMM-European School of Molecular Medicine, University of Naples, “Federico II”, 80131 Naples, Italy; 5 Department of Public Health, University of Naples “Federico II”, Via S. Pansini, 5, 80131 Naples, Italy; 6 Institute of Experimental Endocrinology and Oncology, Italian National Council of Research, Via S. Pansini 5, 80131 Naples, Italy

**Keywords:** Epigenetics, DNA methylation, Gene regulation

## Abstract

FXYD1 is a key protein controlling ion channel transport. FXYD1 exerts its function by regulating Na^+^/K^+^-ATPase activity, mainly in brain and cardiac tissues. Alterations of the expression level of the FXYD1 protein cause diastolic dysfunction and arrhythmias in heart and decreased neuronal dendritic tree and spine formation in brain. Moreover, FXYD1, a target of MeCP2, plays a crucial role in the pathogenesis of the Rett syndrome, a neurodevelopmental disorder. Thus, the amount of FXYD1 must be strictly controlled in a tissue specific manner and, likely, during development. Epigenetic modifications, particularly DNA methylation, represent the major candidate mechanism that may regulate Fxyd1 expression. In the present study, we performed a comprehensive DNA methylation analysis and mRNA expression level measurement of the two Fxyd1 transcripts, Fxyd1a and Fxyd1b, in brain and heart tissues during mouse development. We found that DNA methylation at Fxyd1a increased during brain development and decreased during heart development along with coherent changes in mRNA expression levels. We also applied ultra-deep methylation analysis to detect cell to cell methylation differences and to identify possible distinct methylation profile (epialleles) distribution between heart and brain and in different developmental stages. Our data indicate that the expression of Fxyd1 transcript isoforms inversely correlates with DNA methylation in developing brain and cardiac tissues suggesting the existence of a temporal-specific epigenetic program. Moreover, we identified a clear remodeling of epiallele profiles which were distinctive for single developmental stage both in brain and heart tissues.

## Introduction

Fxyd domain-containing transport regulator 1 (*Fxyd1)* gene encodes phospholemman (PLM), a small, single-spanning membrane protein that controls cell excitability by modulating Na^+^/K^+^-ATPase activity^1^. Fxyd1 is expressed predominantly in cardiac and skeletal muscle and, to some extent, in the brain^1^. In heart of rats overexpressing endogenous FXYD1, a decrease in Na^+^/K^+^-ATPase current has been reported while Fxyd1-knockout mice exhibit increased cardiac mass, larger cardiac myocyte cross-sectional area, and higher ejection fraction^2,3^. Accordingly, in myocytes, Fxyd1 expression levels drastically increase after myocardial infarction and heart failure^2–4^**.** Thus, the amount of FXYD1 in cardiac tissue must be strictly regulated in order to ensure accurate heart functioning. In comparison, the role of FXYD1 in the brain is less understood. In the brain, merely observing, Fxyd1 expression levels follow a specific regional distribution, being higher in cerebellum (CB) and lower in frontal cortex (FC)^5^. Lower Fxyd1 transcript levels in FC area correlates with higher DNA methylation at Fxyd1 gene^5,6^. DNA methylation may control Fxyd1 expression in brain through the recruitment of the MeCP2 protein, a methyl-binding protein implicated in the regulation of gene expression by binding 5-methyl cytosine modification on promoter region of its target genes^5,6^. Also the binding of MeCP2 and its role on the Fxyd1 promoter follow a region-specific pattern in the brain: in MeCP2-null mice, Fxyd1 mRNA levels increase in the frontal cortex (FC), but not in the cerebellum (CB), indicating that MeCP2 activity in repressing Fxyd1 expression is limited to the FC area^5,6^. Moreover, in the brain, Fxyd1 gene undergoes alternative splicing generating different transcripts. The main transcripts, named Fxyd1a and Fxyd1b, result from the use of different transcription start sites (TSS), being Fxyd1b located downstream the Fxyd1a TSS^5^. Accordingly, Fxyd1a and Fxyd1b transcripts present different putative promoters. Both Fxyd1a and Fxyd1b mRNA levels are more abundant in CB compared to FC, and this difference is more pronounced for Fxyd1b^5^.

Functionally, by interacting with key modulators such as protein kinase A (PKA), protein kinase C (PKC), myotonic dystrophy protein kinase (DMPK), and never in mitosis (NIMA) kinase, FXYD1 is involved in the modulation and maintenance of neural excitability^7–12^. Thus, considering its role in regulating such a key function in neurons, a further detailed understanding of the expression levels of Fxyd1 and its different transcripts in the brain is actually needed. Critically, at our knowledge no studies have to date investigated the expression dynamics of the two Fxyd1 transcripts in brain and, per se and by comparison, in heart during development. Thus, in the present study, we performed a comprehensive DNA methylation analysis and mRNA expression level measurement of Fxyd1a and Fxyd1b in brain and heart tissues of mice at post-natal day 1 (P1), post-natal day 15 (P15) and post-natal day 60 (P60) to evaluate whether promoter-specific DNA methylation may control the expression of Fxyd1a and Fxyd1b in brain and cardiac tissues during mouse development. We found that both in brain and in heart Fxyd1a was less methylated and more expressed than Fxyd1b at any developmental stage. Moreover, DNA methylation at Fxyd1a increased during brain development and decreased during heart development in line with changes of mRNA expression levels. We have previously demonstrated that ultra-deep methylation analysis may help to unravel cell-to-cell heterogeneity in terms of DNA methylation^13–17^. By using a newly generated bioinformatic tool^18^, we were able to assess the quantitative and qualitative distribution of epialleles, intended as combination of methylated and unmethylated CpG sites on the same molecule. Such epiallele analysis, here applied as a proxy of single cell analysis, allowed us to identify distinct methylation profile distributions between heart and brain and in different developmental stages. Different CpG methylation arrangements were detected by epiallele analysis even among cases in which the average methylation was the same. Our data strongly suggest that the transcription of Fxyd1 gene and its two isoforms is regulated by a temporal-specific epigenetic program involving DNA methylation both in brain and in cardiac tissues. Moreover, we identified a clear remodeling of epialleles profiles which were distinctive for single developmental stages both in brain and heart tissues.

## Results

### DNA methylation at Fxyd1a and Fxyd1b promoters inversely correlate with the respective transcripts level at the different stages of brain development

**Figure 1:**
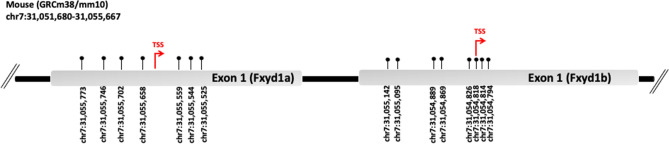
Schematic representation of Fxyd1 gene. Promoters of Fxyd1a and Fxyd1b isoforms are shown. The chromosomal coordinates of the entire Fxyd1 are reported. The two alternative Transcriptional Start Sites are indicated with red arrows. Position of each analyzed CpG is indicated with the respective genomic coordinates.

**Figure 2:**
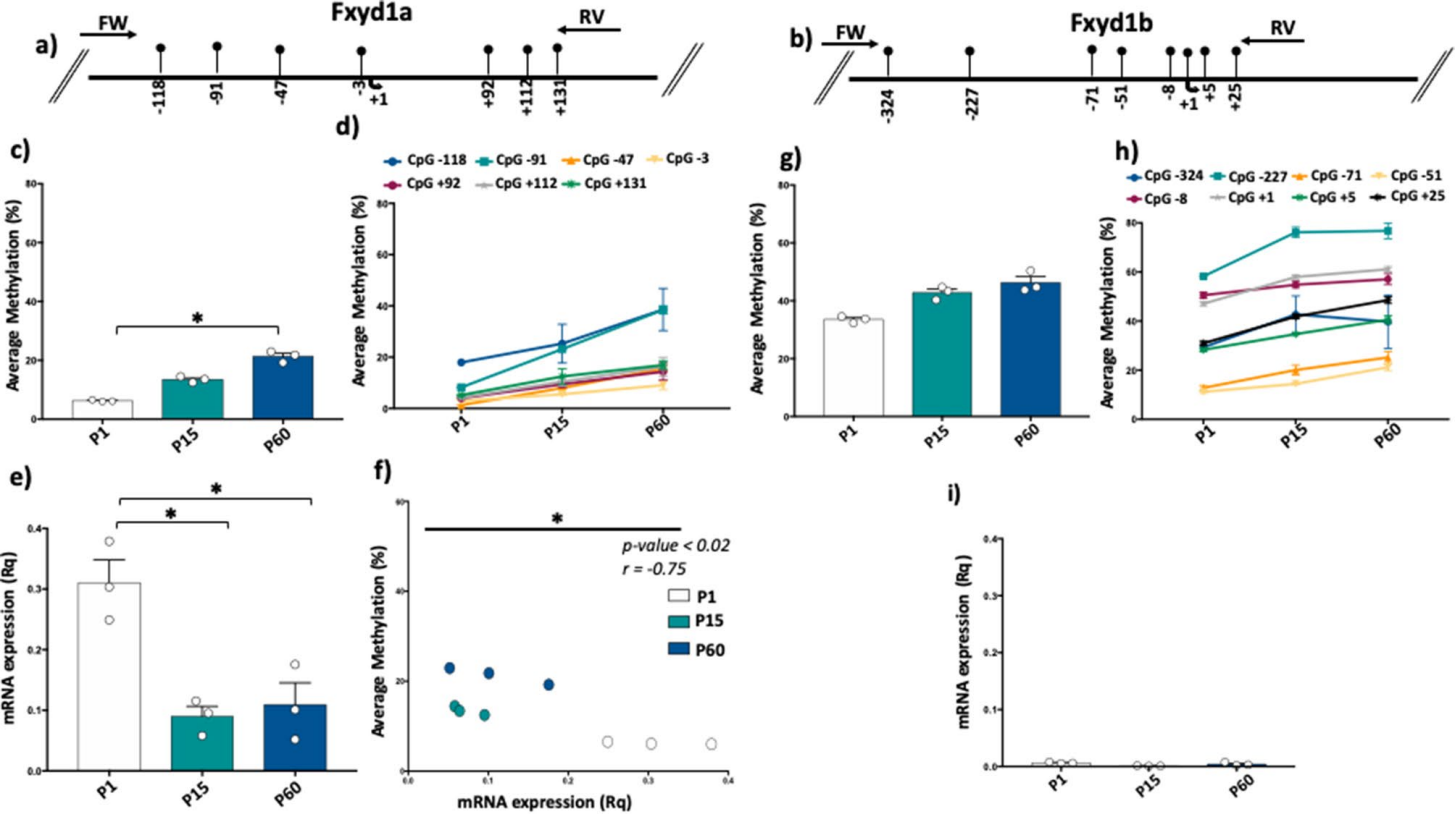
DNA methylation and mRNA expression of the Fxyd1 isoforms in the brain. (**a**) Fxyd1a gene promoter structure showing the analyzed CpGs. Seven CpG sites were analyzed at Fxyd1a promoter. The numbers of the CpGs are referred to the transcription starting site (TSS) indicated with the black arrow at + 1. Black arrows at the top specify the position of the primers used for bisulfite amplification. (**b**) Fxyd1b gene promoter structure showing the analyzed CpGs. Eight CpG sites were analyzed at Fxyd1b gene. The numbers of the CpGs refer to the transcription starting site (TSS) indicated with the black arrow at + 1. Black arrows at the top specify the position of the primers used for bisulfite amplification. (**c**) Fxyd1a average methylation (%) showed at P1, P15 and P60 developmental stages. Comparisons between developmental stages were performed using One-way ANOVA followed by Multiple t test. (**d**) Average methylation at single CpG sites displaying the methylation trend in the different developmental stages at Fxyd1a promoter. (**e**) mRNA expression levels of Fxyd1a referred to each developmental stage. Fxyd1a mRNA expression was normalized to the mean of the housekeeping gene and is expressed as 2^–ΔCt^ values. Comparisons between developmental stages were performed using One-way ANOVA followed by Multiple t test. (**f**) Pearson correlation between Fxyd1a mRNA expression and Fxyd1a promoter methylation at all analyzed developmental stages. (**g**) Fxyd1b average methylation (%) is showed at P1, P15 and P60 developmental stages. (**h**) Average methylation at each of analyzed CpG sites at Fxyd1b locus at the different developmental stages. (**i**) Expression levels of Fxyd1b referred to each developmental stage. Fxyd1b mRNA expression was normalized to the mean of the housekeeping gene and is expressed as 2^–ΔCt^ values. Comparisons between developmental stages were performed using One-way ANOVA followed by Multiple t test.*p < 0.05.

We first analyzed DNA methylation at the promoter region of both Fxyd1a and Fxyd1b transcripts in brain of mice at P1, P15, and P60. The two different isoforms of Fxyd1 gene and the genomic coordinates of analyzed CpG sites were reported (Fig. 1). At Fxyd1a promoter, we analyzed a region of 388 bp containing 7 CpG sites encompassing the Transcriptional Start Site (TSS) (Fig. 2a). For Fxyd1b transcript, DNA methylation was analyzed at a region of 403 bp containing 8 CpG sites surrounding the TSS (Fig. 2b). Both regions were previously analyzed and identified as target of MeCp2 protein^5,6^. First, we found that Fxyd1a promoter showed a lower degree of methylation compared to Fxyd1b for all analyzed developmental stages (Fig. 2c,g). Moreover, a significant increase (p-value = 0.011, One-way ANOVA, followed by Multiple T test) of DNA methylation at Fxyd1a promoter was found during brain development, specifically comparing P1 and P60 (Fig. 2c). Conversely, no significant changes were observed at Fxyd1b transcript during time (Fig. 2g). We also evaluated DNA methylation levels at each of the single CpG analyzed at both Fxyd1a and Fxyd1b (Fig. 2d,h). We found increasing DNA methylation levels during time at all CpG sites at both transcript regulatory regions. More in detail, CpG -118 and CpG -91 at Fxyd1a presented the highest levels of DNA methylation at all analyzed developmental stages (Fig. 2d), especially CpG -91 that significantly (One-way ANOVA, followed by Multiple T test; p-value < 0.0001) increased in DNA methylation overtime, ranging from 8.2% ± 0.23 (mean ± standard error) of methylation at P1 to 38.5% ± 1.5 (mean ± standard error) at P60 (Fig. 2d). Similarly, at Fxyd1b promoter, a trend of increasing DNA methylation was observed during brain development at all analyzed CpG sites (Fig. 2h). Particularly, CpG -227 showed the highest level of DNA methylation in all analyzed stages and a strong and significant increase (One-way ANOVA, followed by Multiple T test; p-value = 0.0016) of DNA methylation from P1 (58.22 ± 0.14) to P15 (76.14 ± 2.13) (Fig. 2h). We then analyzed mRNA expression at both Fxyd1 transcripts at P1, P15 and P60 (Fig. 2e,i). In accordance with the higher level of DNA methylation, Fxyd1b mRNA expression was very low compared to Fxyd1a mRNA levels at any of the analyzed stages (Fig. 2e,i). Dynamically, we observed a significant decrease in mRNA expression of Fxyd1a transcript from P1 to P15 (P1 vs P15, p-value = 0.02; P1 vs P60, p-value = 0.02, One-way ANOVA, followed by Multiple T test) (Fig. 2e) while very low levels of Fxyd1b transcript mRNA were detected at all analyzed stages, with no significant differences among time points (Fig. 2i). Thus, we correlated the DNA methylation degree with the level of mRNA expression during brain development at Fxyd1a (Fig. 2f). We performed correlation analysis only at Fxyd1a promoter, considering the low levels of mRNA expression of Fxyd1b. Intriguingly, we found a significant negative correlation (Pearson Correlation, p-value < 0.02; r = − 0.75) between mRNA expression and DNA methylation average at Fxyd1a, indicating that, during brain development, the changes of Fxyd1a expression are associated with DNA methylation changes detected at the promoter region of this gene (Fig. 2f).

### Fxyd1a and Fxyd1b DNA methylation and mRNA expression scenario in heart during development

**Figure 3:**
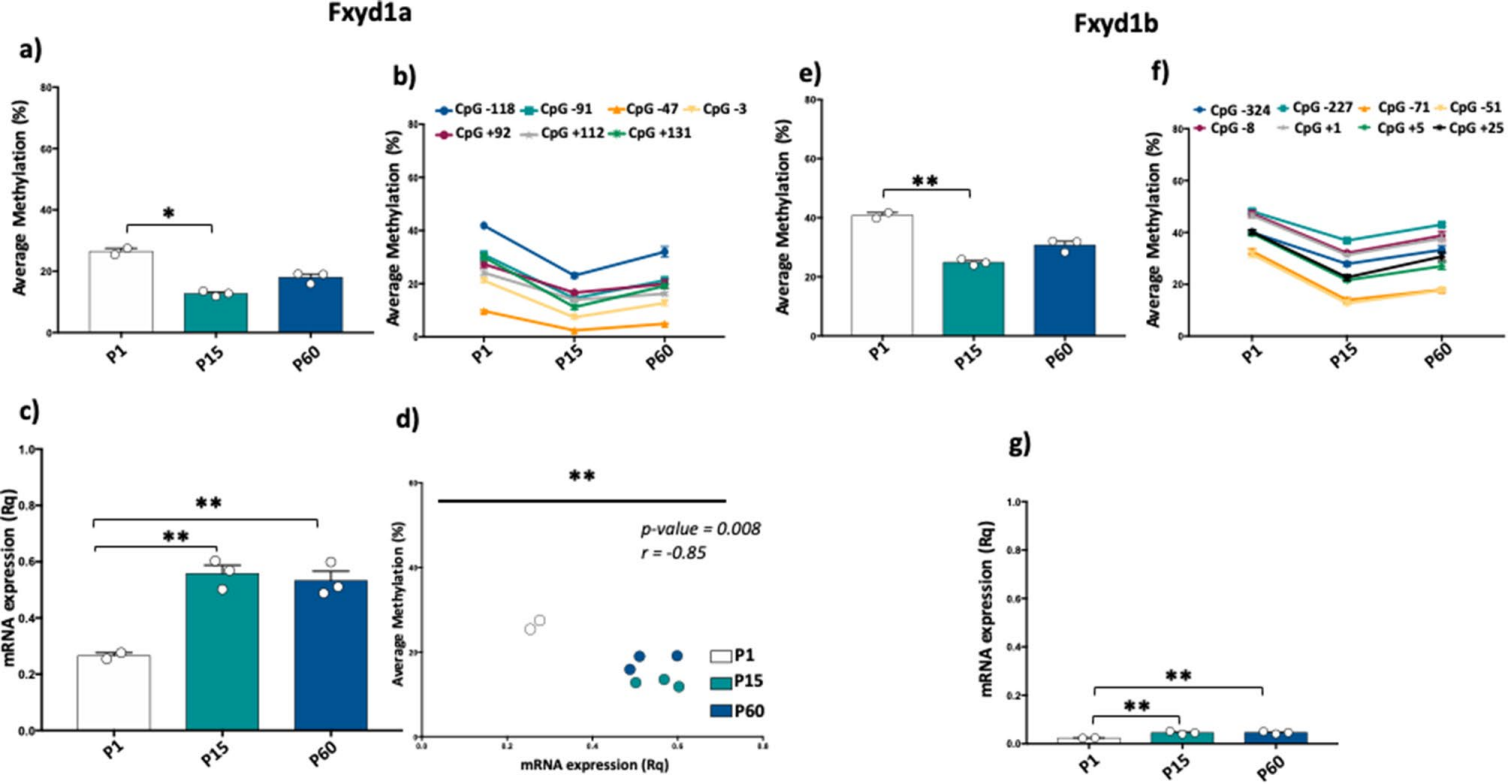
DNA methylation and mRNA expression of the Fxyd1a and Fxyd1b genes in the heart. (**a**) Fxyd1a average methylation (%) at P1, P15 and P60 developmental stages. Comparisons between developmental stages were performed using One-way ANOVA followed by Multiple t test. (**b**) Average methylation at all analyzed CpG sites at Fxyd1a promoter during heart development. (**c**) mRNA expression levels of Fxyd1a at all analyzed developmental stages; Fxyd1a mRNA expression was normalized to the mean of the housekeeping gene and is expressed as 2^–ΔCt^ values. Comparisons between developmental stages were performed using One-way ANOVA followed by Multiple t test. (**d**) Correlation between Fxyd1a mRNA expression and Fxyd1a average methylation during heart development. (**e**) Fxyd1b average methylation (%) showed at P1, P15 and P60 developmental stages. Comparisons between developmental stages were performed using One-way ANOVA followed by Multiple t test. (**f**) Average methylation at single CpG sites indicating the methylation trend in the different developmental stages at Fxyd1b locus. (**g**) mRNA levels of Fxyd1b referred to each developmental stage; Fxyd1b mRNA expression was normalized to the mean of one housekeeping gene and is expressed as 2^–ΔCt^ values. Comparisons between developmental stages were performed using One-way ANOVA followed by Multiple t test. *p < 0.05; **p < 0.01.

We then performed DNA methylation analysis and mRNA expression measurement in heart tissue of mice at P1, P15 and P60. Similarly to the brain, DNA methylation levels were higher at Fxyd1b promoter compared to Fxyd1a in all analyzed groups (Fig. 3a,e). In detail, we found a significant decrease of Fxyd1a DNA methylation at P1 compared to P15 (p-value = 0.017; One-way ANOVA, followed by Multiple T test) (Fig. 3a). Furthermore, Fxyd1b promoter methylation was significantly higher at P1 compared to P15 (p-value = 0.003; One-way ANOVA, followed by Multiple T test) (Fig. 3e). The robust decrease in DNA methylation from P1 to P15 was observed also at all analyzed CpG sites at both Fxyd1a and Fxyd1b (Fig. 3b,f). For Fxyd1a, this phenomenon occurred especially at CpG − 118, the more methylated CpG site overtime, that underwent the strongest and significant decrease in DNA methylation from P1 to P15 and P60 (P1 vs P15: p-value = 0.001, P1 vs P60: p-value = 0.02; One-way ANOVA, followed by Multiple T test) (Fig. 3b). At Fxyd1b promoter, a strong a significant decrease of DNA methylation from P1 to P60 was found at CpG − 71 (P1 vs P15: p-value < 0.0001, P1 vs P60: p-value = 0.0002; One-way ANOVA, followed by Multiple T test) and CpG -51 (P1 vs P15: p-value < 0.0001, P1 vs P60: p-value = 0.0002; One-way ANOVA, followed by Multiple T test) that were also the two CpG sites with the lowest levels of DNA methylation during heart development. As we observed in brain, Fxyd1a transcript was overtime more expressed compared to Fxyd1b in heart tissue (Fig. 3c,g). Particularly, for Fxyd1a, we observed a significant increased mRNA expression at P15 and P60 compared to P1 (P1 vs P15, p-value = 0.003; P1 vs P60, p-value = 0.004, One-way ANOVA, followed by Multiple T test) (Fig. 3c). Despite the lower mRNA expression compared to Fxyd1a, also Fxyd1b transcript showed a significant increase of expression at P15 and P60 compared to P1 (P1 vs P15, p-value = 0.0099; P1 vs P60, p-value = 0.0093, One-way ANOVA, followed by Multiple T test) (Fig. 3g). Therefore, we evaluated whether also in heart tissue DNA methylation at Fxyd1a correlated with their expression during development (Fig. 3d). We found a significant correlation between average methylation and mRNA expression at Fxyd1a in heart during development (Pearson Correlation, p-value = 0.008; r = − 0.85). Thus, we conclude that in heart, as well in the brain, DNA methylation associates during time with the expression of Fxyd1 gene transcripts during development.

### Epiallele classes and epiallele distribution analyses at Fxyd1a and Fxyd1b highlight specific methylation signatures during brain and heart development

**Figure 4:**
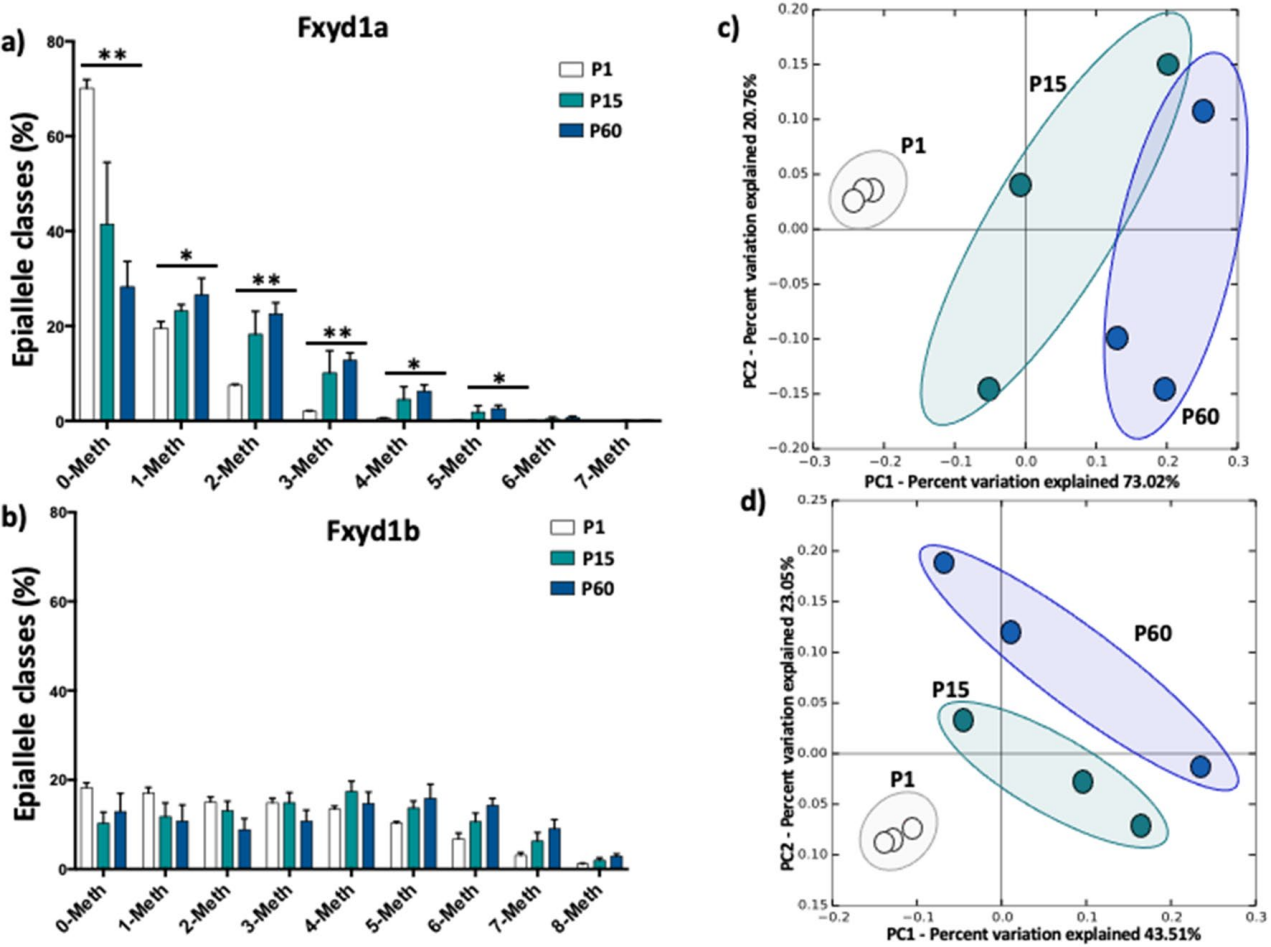
Epiallele classes and epiallele distribution analyses at the Fxyd1a and Fxyd1b in the brain. (**a**) Epiallele classes distribution of Fxyd1a promoter. The percentage of each epiallele class is shown for all analyzed developmental stages. Epiallele classes amount at each developmental stage was obtained by the sum of all epialleles carrying the same number of methylated CpG sites, regardless the position. Comparison between developmental stages was performed using One-way ANOVA. (**b**) Epiallele classes distribution of Fxyd1b promoter. Epiallele classes percentage is shown for all developmental stages. Epiallele classes amount at each developmental stage was obtained by the sum of all epialleles carrying the same number of methylated CpG sites, regardless the position. Comparison between developmental stages was performed using One-way ANOVA (**c**) Principal Component Analysis (PCA) plot showing epialleles distribution at Fxyd1a in brain at all the different developmental stages (white: post-natal day 1; green: post-natal day 15; blue: post-natal day 60). The analysis was based on all the possible epiallele combinations given by the number of analyzed CpGs (128 possible epialleles). PCA was performed by running the beta_diversity_through_plots.py script from QIIME. The Principal Components 1 and 2, ranked according to the fraction of between sample variance explained, are used to plot the samples in the bidimensional space. The two components capture more than 50% of the variance of the data. (**d**) PCA plot showing epialleles distribution at Fxyd1b in brain between the different developmental stages (white: post-natal day 1; green: post-natal day 15; blue: post-natal day 60). The analysis was based on all the possible epiallele combinations given by the number of analyzed CpGs (256 possible epialleles). PCA was performed by running the beta_diversity_through_plots.py script from QIIME. The Principal Components 1 and 2, ranked according to the fraction of between sample variance explained, are used to plot the samples in the bidimensional space. The two components capture more than 50% of the variance of the data. *p < 0.05; **p < 0.01.

**Figure 5:**
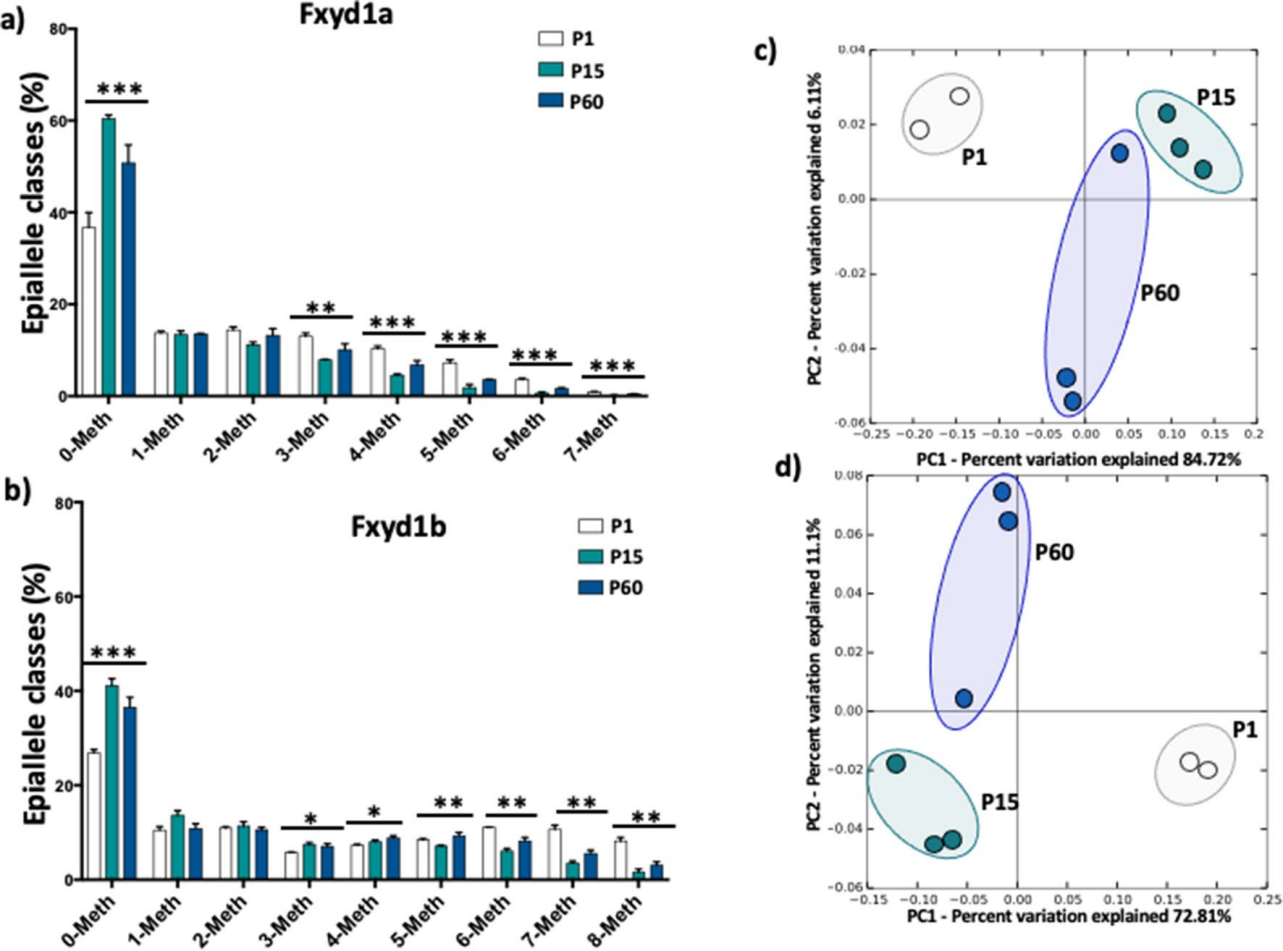
Epiallele classes and epiallele distribution analyses at the Fxyd1a and Fxyd1b in the heart. (**a**) Epiallele classes distribution of Fxyd1a promoter. The percentage of each epiallele class is shown for all analyzed developmental stages. Epiallele classes amount at each developmental stage was obtained by the sum of all epialleles carrying the same number of methylated CpG sites, regardless the position. Comparison between developmental stages was performed using One-way ANOVA. (**b**) Epiallele classes distribution of Fxyd1b promoter. Epiallele classes percentage is shown for all developmental stages. Epiallele classes amount at each developmental stage was obtained by the sum of all epialleles carrying the same number of methylated CpG sites, regardless the position. Comparison between developmental stages was performed using One-way ANOVA (**c**) Principal Component Analysis (PCA) plot showing epialleles distribution at Fxyd1a in brain at all the different developmental stages (white: post-natal day 1; green: post-natal day 15; blue: post-natal day 60). The analysis was based on all the possible epiallele combinations given by the number of analyzed CpGs (128 possible epialleles). PCA was performed by running the beta_diversity_through_plots.py script from QIIME. The Principal Components 1 and 2, ranked according to the fraction of between sample variance explained, are used to plot the samples in the bidimensional space. The two components capture more than 50% of the variance of the data. (**d**) PCA plot showing epialleles distribution at Fxyd1b in brain between the different developmental stages (white: post-natal day 1; green: post-natal day 15; blue: post-natal day 60). The analysis was based on all the possible epiallele combinations given by the number of analyzed CpGs (256 possible epialleles). PCA was performed by running the beta_diversity_through_plots.py script from QIIME. The Principal Components 1 and 2, ranked according to the fraction of between sample variance explained, are used to plot the samples in the bidimensional space. The two components capture more than 50% of the variance of the data.*p < 0.05; **p < 0.01; ***p < 0.001.

**Figure 6:**
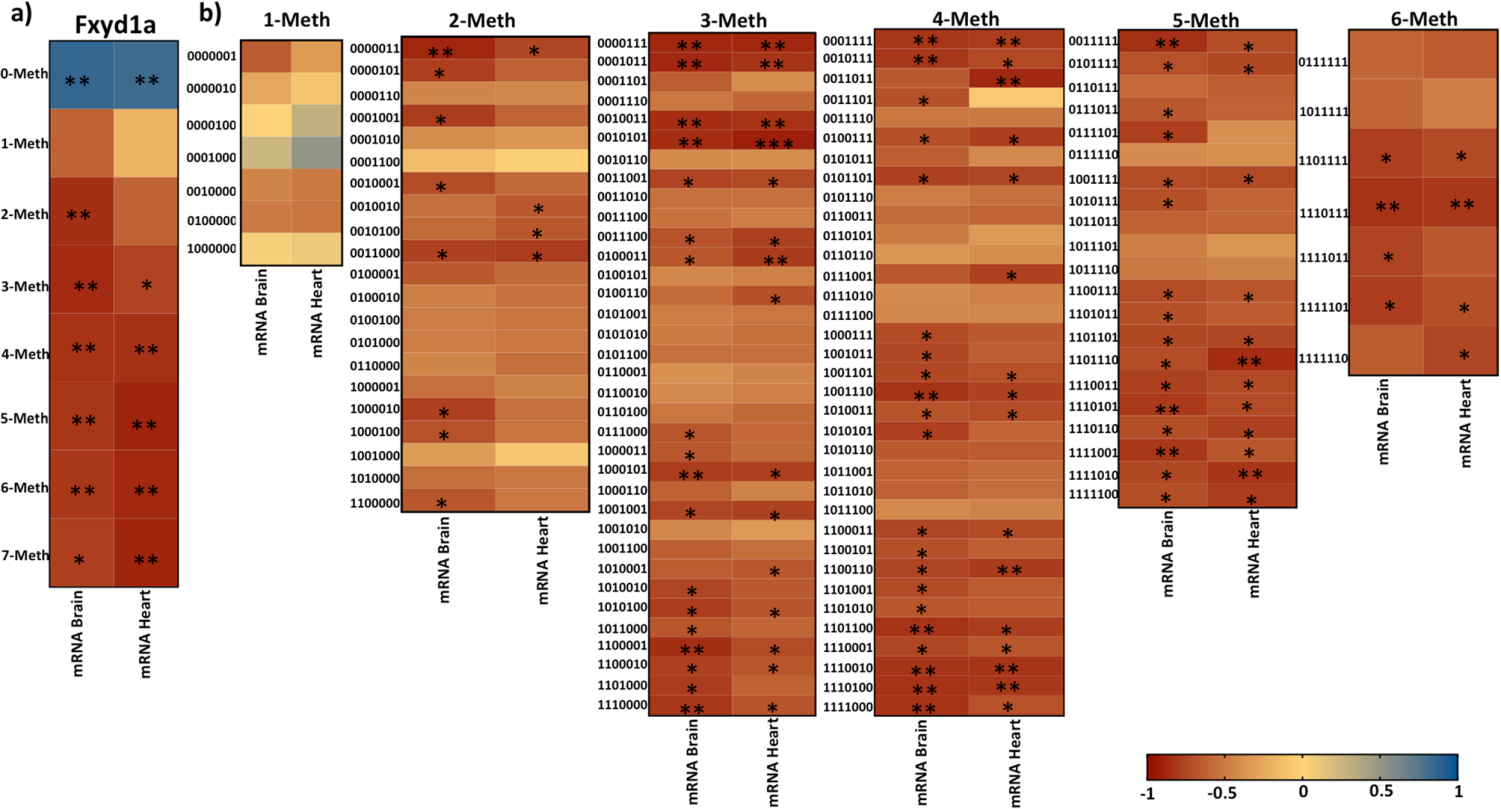
Correlation between epiallele classes and epiallele distribution with mRNA expression levels of Fxyd1a in brain and heart. (**a**) The graph reports r values derived from Pearson correlation between epiallele classes and mRNA expression of Fxyd1a, regardless of developmental time points. (**b**) Heatmaps of r values derived from Pearson correlation between epialleles and mRNA expression of Fxyd1a in brain and heart. Epialleles are shown as a string of numbers where 1 indicates methylated CpG sites and 0 indicates non-methylated CpG sites. The numbers of the strings correspond to the CpG sites analyzed in the Fxyd1a amplicon (shown in Fig. 2a) in the following order: − 118, − 91, − 47, − 3, + 92, + 112, + 131. The scale color from blue to red indicates a positive to negative correlation (− 1 ≤ r ≤ 1), respectively. Statistical analyses were performed using a Pearson correlation test (*p ≤ 0.05; **p ≤ 0.01).

Mammalian tissues are heterogeneous mixture of cells that may possess a different transcriptional program and, consequently, different methylation signatures. Disentangling this phenomenon requires technical approach that may be challenging. In our previous works^13–17^, we demonstrated that the analysis of epiallele distribution may be considered a proxy of single cell studies, since epialleles represent the specific combination of methylated and unmethylated CpGs at single molecule levels. Therefore, evaluating the distribution of epialleles may mirror the different cellular composition in a given tissue and may identify DNA methylation differences among cells. Thus, we decided to investigate the epialleles distribution at Fxyd1a and Fxyd1b in brain during development. We first evaluated the epiallele classes, defined as the sum of molecules carrying the same number of methylated CpG regardless the position on the molecule. We identified significant differences in epiallele classes distribution at Fxyd1a promoter (Fig. 4a). As expected by the increase in average methylation from P1 to P60, we found a significant decrease in 0-Meth class overtime and a significant concomitant increase in 1-, 2-, 3-, 4- and 5-Meth classes during brain development (Fig. 4a). Conversely, Fxyd1b epiallele classes distribution did not change among the different stages (Fig. 4b). We then applied epiallele distribution analysis However, when we applied epiallele distribution analysis, also considering the specific position of methylated CpG sites on the same molecules, we identified striking differences in epiallele composition. We found that Fxyd1a epialleles were able to not only discriminate all the developmental time points but also to identify clear clustering discriminating P15 and P60, despite the average methylation did not significantly change (Fig. 4c). Surprisingly, even sharing the same average methylation and no significant changes in epiallele classes distribution, Fxyd1b epiallele profiles clearly distinguished the different time point during brain development (Fig. 4d). Subsequently, we applied epiallele classes and epiallele distribution analyses in heart development. In this case, we identified a significant increase in 0-Meth class both at Fxyd1a and Fxyd1b promoter (Fig. 5a,b), in line with the decrease in average methylation from P1 to P60. Moreover, we found significant differences in 3-, 4-, 5-, 6- and 7-Meth classes at Fxyd1a and 3-, 4-, 5-, 6-, 7- and 8-Meth classes differences at Fxyd1b (Fig. 5a,b). Deepening the investigation, also in cardiac tissue characteristic epiallele distribution both at Fxyd1a and at Fxyd1b clustered the different developmental stages (Fig. 5c,d), especially distinguishing P15 and P60 that presented a very similar average methylation. Considering the changes in epiallele classes distribution at Fxyd1a both in brain and in heart, we decided to correlate all epiallele classes and epiallele distributions, regardless the developmental stages, with mRNA expression of Fxyd1a in both tissues. We reported the r value resulting from the Pearson correlation in the heatmaps in Fig. 6a,b. We found several positive and negative significant correlations between epiallele classes and mRNA expression of Fxyd1a in both brain and heart. As expected, Fxyd1a mRNA expression positively and significantly correlated with the amounts of 0-Meth classes in both tissues. Moreover, 2-, 3-, 4-, 5-, 6- and 7-Meth classes significantly and inversely correlated with the expression of Fxyd1a in brain. In heart, Fxyd1a expression was negatively correlated with 3-, 4-, 5-, 6- and 7-Meth classes (Fig. 6a). We then correlated the amount of each of the detected epiallele in each class with Fxyd1a expression (Fig. 6b). Each epiallele was shown as a string of 1 and 0, indicating methylated CpG sites and non-methylated CpG sites, respectively. We found several significant negative correlations between epialleles abundance and Fxyd1a mRNA expression in both heart and brain. Interestingly, although the average methylation and mRNA expression of Fxyd1a in brain compared to heart tissues followed opposite trend, the majority of epialleles which abundance correlated with Fxyd1a expression were shared by heart and brain tissues. Thus, in two completely different tissues and considering all the developmental time points, we found that specific arrangements of methylated CpGs significantly correlated to Fxyd1a silencing. These results support the role of DNA methylation in controlling the mRNA expression of Fxyd1a in both brain and cardiac muscle. Additionally, these data suggest that specific arrangements of simultaneously methylated CpG sites, including two non-consecutive CpG sites, was likely sufficient to inactivate the expression in brain, while in cardiac tissue, precise profiles with at least three simultaneously methylated CpG sites, were needed to silence the expression of Fxyd1a (Fig. 6a,b).

### Epiallele distribution at Fxyd1a and Fxyd1b promoters discriminates heart and brain tissues

**Figure 7:**
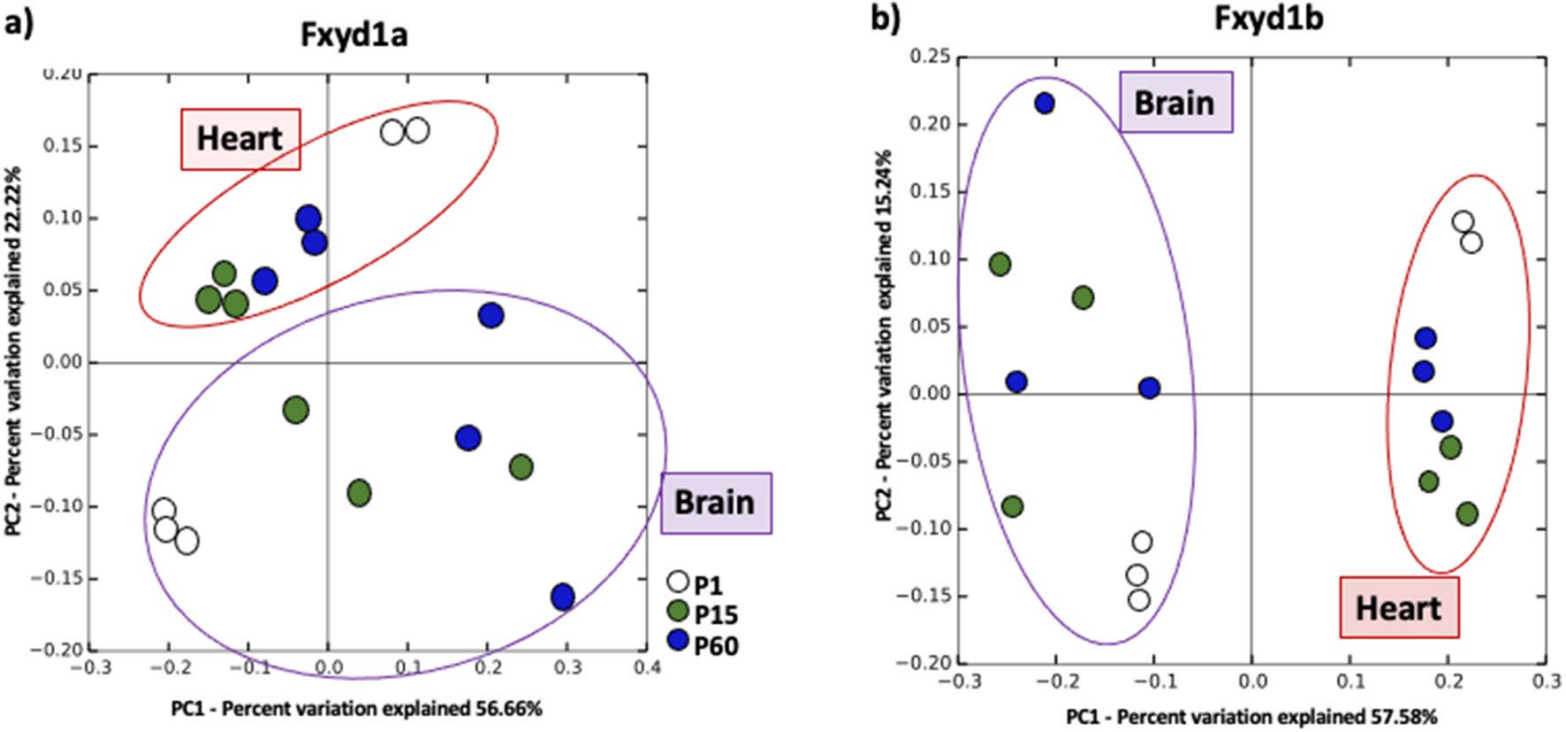
Epialleles distribution of Fxyd1a and Fxyd1b genes in brain and heart performed by PCA. Red and purple circles represent sample clusters based on epiallele distribution in heart and brain, respectively, considering all developmental stages. (**a**) Epialleles distribution analysis of Fxyd1a gene applied on both analyzed tissues pulling together all the developmental stages. (**b**) Epialleles distribution analysis of Fxyd1b promoter applied on both tissues analyzed considering all the developmental stages.

We then applied epiallele distribution analysis to evaluate differences between tissues regardless the developmental stage (Fig. 7a,b). Both at Fxyd1a and Fxyd1b, brain and cardiac tissues clearly clustered away from each other indicating that the epialleles composition was greatly different between the two tissues (Fig. 7a,b). This phenomenon may be quite expected by the fact that we are analyzing completely different tissues. However, the average methylation was almost similar, especially at P15 and P60 at Fxyd1a and P1 at Fxyd1b. Thus, our analysis demonstrated that, by contrast with the simple determination of average methylation, epiallele analysis of Fxyd1 may reveal DNA methylation signatures typifying the different cellular composition and define the cell-to-cell differences of epiallele profiles.

## Discussion

During the early post-natal period, some critical changes in the DNA methylation patterns occur at genes that must undergo to long lasting changes of expression program, in order to allow the correct brain and heart function^13,15,19–24^. Robust data indicate that Fxyd1 product plays a critical role in brain and heart tissues^2–12^. Although changes of Fxyd1 level were observed in MeCP2 KO mice and in the brain of Rett patients^5,6^, poor data are available on the expression and DNA methylation dynamics during the early post-natal period. Thus, in the present study, we analyzed DNA methylation at Fxyd1a and Fxyd1b, two different isoforms of Fxyd1 presenting two different putative promoters, during brain and heart development. We found a strong and significant decrease in the expression of Fxyd1a in brain immediately after birth while in heart a post-natal strong activation must occur to ensure the physiological levels of Fxyd1a. Moreover, in line with a previous work^5^, we found that Fxyd1b is more methylated and less expressed both in brain and in heart, indicating that the major activity of Fxyd1 is due to the isoform Fxyd1a, whose DNA methylation levels changed during brain and heart development. This mechanism may be related to MeCP2 recruitment that, as previously demonstrated^5^**,** binds Fxyd1a promoter, by negatively regulating its expression. Thus, our data suggest that DNA methylation at Fxyd1a promoter increased after birth in the brain, possibly leading to an increase of MeCP2 binding along with a reduced Fxyd1a mRNA expression. Since MeCP2 also binds to Fxyd1b promoter^5^, the absence of change in DNA methylation and mRNA expression at Fxyd1b let us speculate that MeCP2 binds to the Fxyd1b alternative TSS immediately after birth, strongly repressing the transcription of this isoform in developing brain. Conversely, in cardiac tissue decreased DNA methylation and concomitant increased mRNA expression may be due to a detachment of MeCP2 on the Fxyd1a promoter and, at low extent, on the promoter of Fxyd1b. Interestingly, elevated levels of FXYD1, with concomitant reduction of Na^+^/K^+^-ATPase activity, were found in frontal cortex neurons of Rett patients, suggesting a role for FXYD1 in the abnormal neuronal activity in Rett syndrome^5^. Moreover, increased levels of FXYD1 were also found in cortical neurons of MeCP2 deficient mice and the rescue of FXYD1 expression resulted in the partial rescue of the main impairments in MeCP2-null mice^25^. Since the binding of some Methyl Binding Protein (MBP), including MeCP2, may be influenced by a precise arrangement of methylated and unmethylated CpG sites^26,27^, the identification of methylation pattern distribution may be extremely important, especially for those genes that are targets of MBD, such as Fxyd1. The here presented data do not allow us to fully address this point because they do not enable one to associate specific methylation patterns with preferential MeCP2 binding. However, thanks to the high resolution of DNA methylation analysis and the high coverage of sequencing, we here were able to perform a proxy of single-cell analysis, quantifying the single molecule methylation heterogeneity. This analysis considers that each molecule in each cell may present a specific distribution of methylated-CpG sites, defined “epielleles”, and that detected methylation profiles correspond to the configuration of a single allele in a single cell belonging to tissue cell mixture, accounting for cell-to-cell methylation variability. This approach, however, presents two limitations: despite we can identify different mCpG phases at single molecule level, we are no able to distinguish the specific cell types and subtypes bearing a specific epiallele; moreover, a specific functional role to any epiallele arrangement cannot be attributed. Other approach, such as single cell analyses, may greatly contribute to address these questions. By applying epiallele classes and epiallele distribution analyses, we identified methylation signature at Fxyd1a and Fxyd1b promoters that distinguish not only different developmental stages but also different tissues, even when average methylation was the same. Intriguingly, when we correlated the amount of epiallele classes at Fxyd1a with the mRNA expression levels both in brain and in heart, we found that specific epiallele classes may be linked to the silencing of Fxyd1 expression. Our analyses suggest that, for Fxyd1a, specific arrangements of few simultaneously methylated CpG sites were sufficient to silence the expression of Fxyd1a both in brain and in heart. Thus, the repressive binding of MeCP2 on Fxyd1a promoter likely occurs already when few CpG sites are methylated. Thus, our data strongly suggest that the transcription of Fxyd1 gene and its two isoforms is regulated by a temporal-specific epigenetic program involving DNA methylation both in brain and in cardiac tissues. Moreover, we identified a clear remodelling of epialleles profiles which were distinctive for single developmental stages both in brain and heart tissues, potentially reflecting the fine DNA methylation differences at Fxyd1 gene at single cell level in the developing brain and heart.

## Materials and methods

### Mouse tissues collection

The whole brain and heart from mice C57BL/6 at different developmental stages were obtained in collaboration with Biogem S.C.aR.L. The facility is authorized to the use of rodents in biomedical researches by the employed office of the Ministry of Health with ministerial decree n° 12/2016–UT, of September 29th, 2016 and follows the rules of the D.lgs. n° 26–March 4th 2014, the Italian law regulating animals housing and use for experimental purposes. This law follows the European Directive 2010/63/EU—22/09/2010, concerning the protection of animals used for research or other scientific purposes. This study was approved by Regione Campania—acting as Azienda Sanitaria Locale (ASL) Avellino. All the procedures involving animals were carried out in accordance with the ARRIVE Guidelines.

### DNA and RNA extraction

Brain and heart tissues were first pulverized and then split to extract DNA and RNA. DNA was extracted from liquid nitrogen–pulverized mouse tissues using DNeasy Blood & Tissue Kit (Qiagen, Hilden, Germany), according to the manufacturer’s instructions. The quality of DNA was checked using NanoDrop 2000 (Thermo Scientific). DNA was quantified using Qubit 2.0 Fluorometer with the dsDNA broad range assay kit (Invitrogen, Q32850). Total RNA from tissues was extracted using RNeasy mini kit (QIAGEN) following the manufacturer’s instructions. The integrity of the RNA was determined using NanoDrop 2000 (Thermo Scientific). Recombinant DNase (QIAGEN) was used to remove potentially contaminating genomic DNA.

### qRT-PCR

RNA of each sample (1 μg of the extracted RNA) was firstly denatured and then reverse-transcribed using QuantiTect Reverse Transcription kit (QIAGEN) following the manufacturer’s instructions. Real Time-PCR amplifications were performed using LightCycler 480 SYBR Green I Master (Roche Diagnostic) in a LightCycler480 RealTime thermocycler. The following protocol was adopted: 10 s for initial denaturation at 95 °C followed by 40 cycles consisting of 10 s at 94 °C for denaturation, 10 s at 60 °C for annealing, and 6 s for elongation at 72 °C temperature. The following primers were used for mouse Fxyd1a and Fxyd1b cDNA amplifications: Fxyd1a: FW 5ʹ-GGGACAGCGTGAATGGGAT-3ʹ; RV: 5ʹ-GAGTCAGCCAGGGTCAAGAA-3ʹ; Fxyd1b: FW: 5ʹ-AGAGAGACCACTGGTTGAGATCCT 3ʹ; RV: 5ʹ CAGCCAGGGTCAAGAAATGT 3ʹ. Actin was used as housekeeping gene for Real-Time PCR and the following primers pair was used: Actin: FW: 5ʹ-CCTCTATGCCAACACAGTGC-3ʹ; RV: 5-CCTGCTTGCTGATCCACATC-3.

### Bisulfite conversion and amplicon library preparation

Bisulfite conversion was performed using EZ DNA Methylation Kit (Zymo Research). Genomic DNA (1 μg) was converted following the manifacturer’s instructions and eluted in 50 μl of H2O. Bisulfite converted DNA underwent to a double-step PCR strategy to generate an amplicon library which was finally sequenced by Illumina Miseq Sequencer. In the first PCR step, bisulfite-specific primers pairs were used: Fxyd1a FW: 5ʹ-TatgTtgTtTTtgggaTtgtgTt-3ʹ; Fxyd1a RV: 5ʹ-ctctcctccctcttAAAtcaAAc-3ʹ; Fxyd1b FW: 5ʹ-gtgtaTTtgtaTataaatgtgtTtg-3ʹ; Fxyd1b RV: 5ʹ- ccaAtAAtctctctAtacccaA-3ʹ. The primers used in the first step of amplification allowed us to analyze a region of 388 bp for Fxyd1a and 403 bp for Fxyd1b encompassing their respective transcription start site and including their putative promoters. The choice of the regions to be analyzed was based on the position of TSSs and on previously reported experiments demonstrating that these regions are bound by MeCP2^5,6^. To estimate the rate of bisulfite conversion, fully unmethylated M13mp18 double-strand DNA (New England BioLabs) was added in representative samples and amplified with the following primers: M13mp18 FW: 5ʹ- Ggtgaagggtaattagttgttgtt-3ʹ; M13mp18 RV: 5ʹ-ccaataccaaacttacatacct-3ʹ. The capital letters in the primer sequences indicate the original C or G. Reactions were performed using FastStart High Fidelity PCR System (Roche) adopting the following protocol: 3 μL 10 × reaction buffer, 0.6 μL of 10 mM dNTP mix, 0.9 μL of 5 mM forward and reverse primers, 3.6 μL MgCl2 25 mM, 4 μL bisulfite template DNA, 0.25 μL FastStart Taq, and H2O up to the final volume (30 μL of final volume). The following thermo-cycle condition was applied: one cycle at 95 °C for 2 min followed by 36 cycles at 95 °C for 30 s, 56 °C for 40 s, 72 °C for 50 s, followed by a final extension step at 72 °C for 6 min. Multiplexing indices and Illumina sequencing adaptors were added at the extremities of first amplicons by performing a second PCR step with the following protocol: 5 μL 10 × reaction buffer, 1 μL dNTP mix, 5 μL forward and reverse “Nextera XT” primers (Illumina, San Diego, CA), 6 μL 25 mM MgCl2, 5 μL of first PCR product, 0.4 μL FastStart Taq, and H2O up to the final volume; 95 °C for 2 min followed by 8 cycles at 95 °C for 30 s, 55 °C for 40 s, 72 °C for 40 s, followed by a final extension step at 72 °C for 5 min. Both PCR amplifications were followed by a purification step with AMPure purification magnetic Beads (Beckman-Coulter, Brea, CA), following the manufacturer’s protocol. Amplicons were then quantified using Qubit 2.0 Fluorometer with the dsDNA broad range assay kit (Invitrogen, Q32850) and diluted to final concentration 4 nM. Amplicons were then pooled and diluted reaching a final concentration of 8 pM. Phix control libraries (Illumina) were combined with normalized library [15% (v/v)] to increase diversity of base calling during sequencing. Amplicon library underwent to sequencing using V2 reagents kits on Illumina MiSeq system (Illumina). Paired-end sequencing was performed in 251 × 2 cycles. An average of 100,000 reads/sample was obtained.

### Sequence handling and bioinformatics analyses

Paired-end reads obtained from Illumina Miseq platform were first assembled using PEAR tool^28^ with a minimum of 40 overlapping residues as threshold. After, FASTQ assembled reads were converted in a FASTA format using PRINSEQ tool^29^. The sequencing information including details on the number of reads per sample, the number of reads obtained after assembling, the analyzed reads and the bisulphite efficiency were summarized in the Supplementary Table 1*.* The obtained sequences were analyzed using ampliMethProfiler pipeline software (https://sourceforge.net/projects/amplimethprofiler)^18^, specifically designed for deep-targeted amplicon bisulfite sequencing. AmpliMethProfiler produces quality filtered FASTA files for each sample and directly extracts methylation average values and methylation profiles. AmpliMethProfiler also generates a tabular format file (BIOM format) containing the number of methylation profiles (epialleles) for all samples. The BIOM table was normalized for the same number of sequence/sample using a rarefaction procedure with QIIME^30^. Principal Component Analysis (PCA) was conducted by running the *beta_diversity_through_plots.py* script from QIIME.

### Statistical analysis

Methylation average data are expressed as mean ± standard error. Comparisons between groups were carried out using one-way ANOVA (with α significance level ≤ 0.05), followed by Multiple T test, and using Pearson Correlation. The mRNA expression levels are reported as 2^−ΔCt^ and analyzed by one-way ANOVA. All statistical analyses were performed using GraphPad Prism version 7.0 (GraphPad Prism Software, Inc., La Jolla, CA, USA www.graphpad.com/guides/prism/7/statistics/index.html).

## Supplementary Information

Supplementary Table 1.

## Data Availability

The datasets generated during the current study are available in the European Nucleotide Archive (ENA) repository, accession number: PRJEB50015.
